# Age Differences in Neural Activity during Slot Machine Gambling: An fMRI Study

**DOI:** 10.1371/journal.pone.0049787

**Published:** 2012-11-28

**Authors:** Anna C. McCarrey, Julie D. Henry, William von Hippel, Gabrielle Weidemann, Perminder S. Sachdev, Michael J. A. Wohl, Mark Williams

**Affiliations:** 1 Department of Clinical Neurosciences, Cambridge University and Medical Research Council Cognition and Brain Sciences Unit, Cambridge, United Kingdom; 2 School of Psychology, University of Queensland, St Lucia, Queensland, Australia; 3 School of Psychology, University of Western Sydney, Penrith, Australia; 4 Brain and Ageing Research Program, School of Psychiatry, University of New South Wales, Sydney, Australia; 5 Neuropsychiatric Institute, Prince of Wales Hospital, Sydney, Australia; 6 Department of Psychology, Carleton University, Ottawa, Ontario, Canada; 7 Macquarie Centre for Cognitive Science, Macquarie University, Sydney, Australia; University G. D'Annunzio, Italy

## Abstract

This study aimed to assess the potential association between age-related prefrontal brain changes and slot machine gambling, an activity that has become increasingly popular among older adults. Functional magnetic resonance imaging was used to assess healthy older and younger adults whilst playing a slot machine. Results revealed that the older group over-recruited several bilateral and contralateral brain structures relative to the younger group. Specifically, older adults exhibited increased neural activation in the superior prefrontal cortex and left orbitofrontal cortex, indicating greater reliance on these structures. These results suggest a compensatory mechanism, by which older adults recruit a greater number of neural networks from both hemispheres to complete the same gambling task as their younger peers. The broader implications of these findings are discussed in relation to theories of neurocognitive and degenerative change that occurs in late adulthood.

## Introduction

Slot machines are generally viewed as the most addictive form of gambling [1]. The rapid playing speed, flickering lights, and “winning” sounds provide an unbroken focal point for attentional capture [1], [2], [3], [4]. As a result, the slot machine player can lose track of time and space in a manner that negatively affects higher order cognitive functioning [5], [6], [7]. Consistent with such a possibility, an increasing number of studies suggest gambling is associated with reductions in executive control [8], [9], [10]. Moreover, problem gamblers are apt to show neural dysfunction in frontal regions [11], [12], [13]. Specifically, activation abnormalities of the prefrontal cortex (PFC) have been observed in problem gamblers during presentation of gambling cues [14] and performance on the Stroop task [15], and medial frontal gyrus activation has been associated with the Iowa Gambling Task [16], [17]. However, further fMRI studies are needed to examine brain activation while gambling, especially during slot machine play [18].

Given age-related changes in the neuroanatomy and neurochemistry of the brain (with frontal structures being disproportionately affected), associated reductions in executive control may render older adults particularly vulnerable to the stimulation provided by the slot machine [19]. Ageing has a stronger relationship with volumetric reductions in prefrontal grey matter than with any other brain region [20], and these neural changes are thought to cause disturbances in affect, cognition, and behaviour that resemble the dysfunction associated with damage to the frontal cortex [21], [22]. Frontal structures are responsible for higher order control operations such as inhibition, mental flexibility, and planning [23], [24], [25], and age-related deficits in such operations are consistently identified [26], [27], [28]. Yet, the potential association between age-related prefrontal changes and gambling has received little attention. In the current study, we examine possible age differences in prefrontal activation as a result of engagement in slot machine play.

Circumstantial support for possible age differences in prefrontal activation whilst gambling comes from the related field of reward learning, where evidence exists for an association between ageing, poorer reward learning, and *over*-activation of the PFC [29]. These findings suggest that older adults recruit additional PFC resources when carrying out reward-based tasks, despite performing more poorly. The finding that older adults over-recruit brain structures during task performance has typically been interpreted as support for the hemispheric asymmetry reduction in older adults (HAROLD) model [30]. A central tenet of this model is that when engaged in cognitive processing, older adults' prefrontal activity tends to be less lateralised than in younger adults. Or in other words, additional prefrontal neural resources are recruited bilaterally, which could help counteract age-related neurocognitive decline [31]. This over-recruitment has been found in a variety of tasks, including decision-making involving risks. For example, older men who made more conservative decisions during a risk-taking task demonstrated increased right orbitofrontal cortex activity relative to younger men [32], a region widely implicated in risky decision making paradigms [33]. Taken together, these studies suggest that prefrontal activity changes with age, such that greater neural activity is required to generate adaptive outcomes when engaging in risky decisions and behaviours that carry rewards and penalties. However, no study to date has assessed whether age-related differences in prefrontal activation are seen on ecologically valid gambling tasks with real monetary rewards.

Laboratory gambling has been found to be a valid reflection of everyday gambling and to elicit high levels of arousal when participants are allowed to retain any winnings accrued [34], [35]. In the only fMRI study to date that has assessed potential age differences in a gambling-related task with monetary rewards, younger and older adults viewed anticipatory cues denoting amounts that could be gained or lost in the proceeding trial [36]. Age differences were revealed in the anticipation of outcomes, with the older group displaying attenuated anterior insula and medial caudate activity to anticipatory cues preceding losses. While the results of this study highlight important age differences in the anticipation of rewards versus losses, anticipatory events are known to activate different neural networks to those of reward outcomes [37]. Furthermore, the task involved a monetary incentive delay task. Specifically, participants encountered distinct cue – fixation – target – fixation – feedback phases during each trial, before being required to respond upon seeing an abstract shape. A central arousing feature of slot machine play, however, stems from the immediacy of monetary reward. In line with the call for fMRI studies that are coordinated with gambling, we examine prefrontal activity while both young and older adults played a slot machine.

Because problem gamblers have been shown to exhibit neural dysfunction in frontal regions [11], [12], [13], it was important that the majority of both young and older gamblers engage in gambling for recreational purposes only. By limiting recruitment to a recreational gambling sample, any differences in neural activation that emerged between age groups would not be due to a propensity for gambling addiction. It was hypothesised that, in keeping with the HAROLD model [30], older adults would over-recruit several bilateral PFC regions whilst gambling, in comparison to younger adults. To ensure over-recruitment among older adults was a result of slot machine play, we included a baseline assessment of activation and compared to a crosshair.

## Methods

### Participants

This study was approved by written consent from the University of New South Wales Human Research Ethics Advisory Panel (Psychology). Ten older (50% male, mean age = 70.2±1.6; mean years of education = 12.3±2.7) and eight younger right-handed adults (50% male, mean age = 21.6±0.9, mean years of education = 15.0±2.0) were recruited from local gaming venues and newspaper advertisements in Sydney. One older male participant completed one part of the gambling session only. All reported gambling at least once per month and were paid $60 as well as any winnings accrued ($20). Participants were screened for a history of neurological disease, psychiatric illness, mood disorders and head injury. In addition, none reported taking medications that could affect blood-oxygen levels and all participants refrained from ingesting caffeine at least two hours prior to scanning. Young and old did not differ on characteristics known to affect gambling behaviour, including cognitive function as indexed by the Wechsler Abbreviated Scale of Intelligence [38], t(16) = 0.65, p = .528 or gambling severity as indexed by the South Oaks Gambling Scale [39], t(16) = 0.71, p = .487. Additionally, the two groups did not differ in level of weekly household income *t*(16) = 1.73, *p* = .102.

### Gambling task

**Figure 1:**
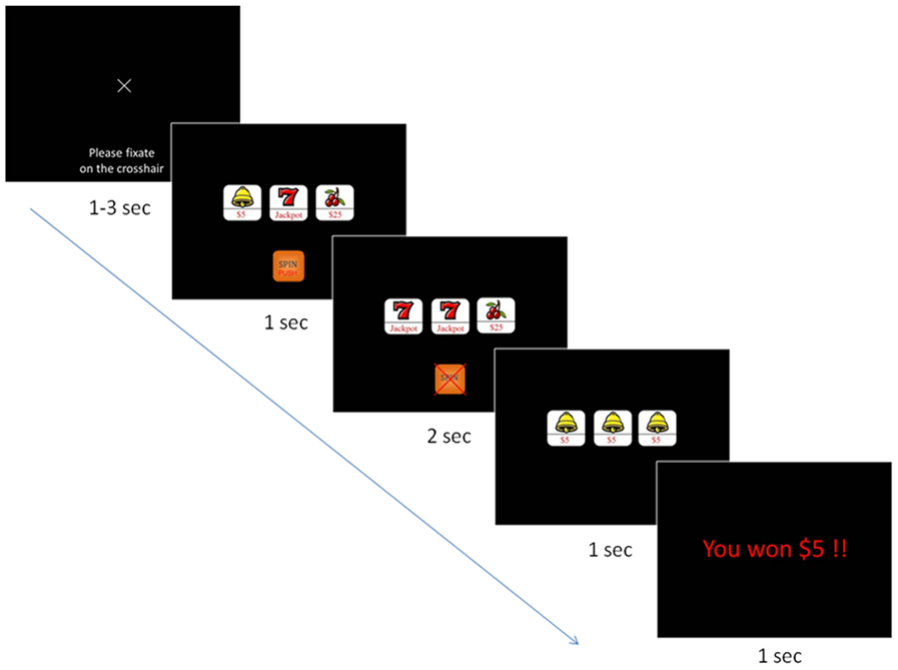
Gambling task for each trial.

A variation of the popular slot machine game ‘Lucky Sevens’ was developed using Presentation® (Version 14.5, www.neurobs.com). This is a simple game whereby three reels spin at the touch of a button. The wager was fixed at $5.00 per spin, and to reduce memory load for gambling outcomes, each icon (e.g. cherry, bell etc.) displayed its monetary value (see Figure 1).

After a jittered 1000–3000 ms baseline period, participants viewed three static icons for 1 s during which time they were required to press a button. The three reels spun at a rate of change of 10 icons per 1000 ms; the first stabilised after 1000 ms, the second after 1500 ms and the third after 2000 ms. Participants viewed the outcome of the trial for 1000 ms followed by a written message of the outcome for 1000 ms. Each run contained 93 spins comprising sequences of wins and losses which were counterbalanced across participants.

### Procedure

Participants were informed that the project had been funded by a ‘casino tax’ that gaming establishments were required to pay in support of gambling research, in a bid to maximise engagement with the task. An open cash box with AU$2000 in various denominations was displayed and it was reiterated that a spin outcome of three sevens would result in a jackpot win of $2000. The cash box remained open throughout a five minute practise session of ‘Lucky Sevens’.

There was no running count on the screen to ensure neural activity did not vary as a function of credits. General feedback regarding performance was provided after every scanning run where each participant learned that they had won money overall in two runs and lost money overall in two runs. These were counterbalanced across participants.

### Image acquisition

Neural activity was measured using a 3-T Philips scanner to acquire T2*-weighted images with BOLD contrast. Functional images were acquired in four runs, each of 341 volumes comprising 38 axial slices of 3 mm thickness and an in-plane resolution of 3 mm. The scanner protocol was as follows: TR = 2000 ms, TE = 30 ms, flip angle = 90°, FOV = 240 mm×240 mm, matrix = 80×80. Images were acquired from a region extending from the prefrontal cortices to the occipital lobes coronally, and from the midpons to the top of the skull axially. This provided optimal spatial resolution for targeted regions of interest while omitting only the medulla, and base of the pons and cerebellum in some participants. The first five volumes were discarded to allow for longitudinal relaxation time equilibrium, and ten volumes (20 s duration) were obtained at task cessation to account for the drift in BOLD signal accrued throughout the session.

### Image pre-processing and analyses

Pre-processing and analyses were conducted using SPM8. Each image was realigned using the first scan as a reference, and the rigid body (six parameters) transformations to best map the series of volumes to the same space were determined [40]. No data were removed due to excessive movement. The mean realigned images were spatially normalised by non-linear transformation to the EPI template supplied by SPM8. The data were fitted to MNI space (Montreal Neurological Institute, Canada) based on the standard stereotaxic coordinate system of Talairach and Tournoux [41] and smoothed with an 8 mm full width at half maximum isotropic Gaussian kernel.

Voxelwise whole brain analysis was used to analyse the regional pattern differences between the two age groups in response to slot machine gambling. Strict criteria were selected to reduce the Type I error rate such that contrasts were thresholded for younger and older adults with a *p* value of .0001 uncorrected, and a 25-voxel extent threshold [42]. Maps were produced for each participant individually for gambling - baseline activity, pooled across all four sessions. Group analyses determined changes in BOLD contrast between age groups during stimulus presentations.

### Brain region selection: Differences in neuro-vascular function

In keeping with prior imaging studies that contrast young and old brain activation [e.g. 32], putative age-related differences in neuro-vasculature that may have impacted upon the interpretation of between-group results were assessed. Beta weight analysis of activity in the precuneus was performed. This brain structure was selected because it, along with neighbouring regions, displays one of the highest resting metabolic rates [43] and while unrelated to the central hypotheses, was extensively activated during the gambling task relative to baseline neural activity. Percent signal changes at significant cluster maxima were calculated and entered into independent *t*-tests to compare neural activation across the age groups.

## Results

### Age differences in neural activation to the gambling task

**Figure 2:**
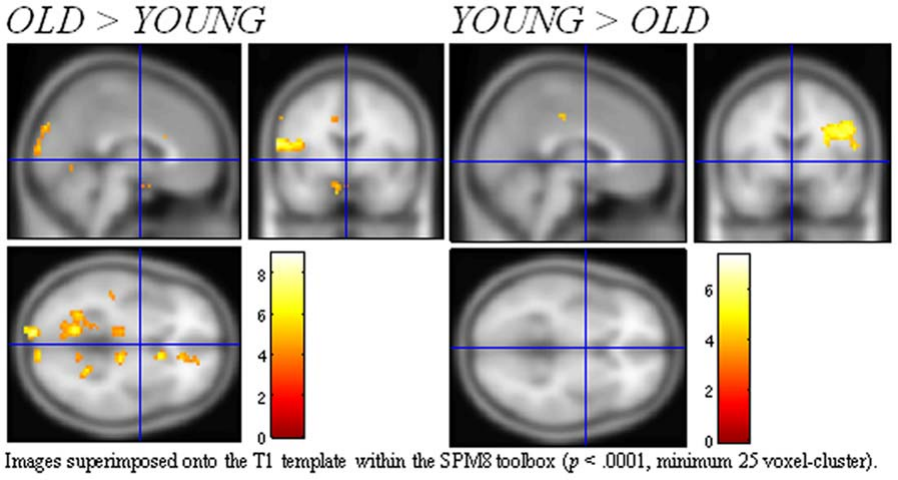
Sagittal, coronal and axial activation t-maps in standard MNI space for both age groups in response to the gambling task.

To isolate neural activity specific to older adults that differed from younger adults, separate analyses were conducted for brain regions activated more by older adults, and brain regions activated more by younger adults. The younger and older adults exhibited different patterns of brain activation in response to the gambling task (Table 1). The younger group recruited more posterior regions such as the occipital gyrus, cerebellum, and angular gyrus, but there were no additional frontal structures activated, the most anterior region being the caudaute nucleus. This is in contrast to the older group, who demonstrated *increased* neural activation relative to the younger group in general, and specifically increased bilateral and contralateral activation in several brain regions including the superior PFC and the orbitofrontal cortex (Figure 2).

**Table 1:** Between group analyses of activation in voxel-clusters in response to the gambling task.

		MNI coordinates			
Region	No. of voxels	*x*	*y*	*z*	*Z score*
*OLD>YOUNG*					
Cuneus	987	9	−100	13	7.19
Precuneus	30	12	−58	49	5.42
Superior temporal gyrus	69	54	−34	16	5.37
Brainstem	67	15	−22	−5	6.59
Motor cortex	589	−63	2	16	6.61
Thalamus	52	−12	−22	7	4.84
Amygdala	123	−9	8	−17	5.41
Cingulate gyrus	25	−12	2	37	4.89
Anterior cingulate	106	15	44	−2	5.26
Sub–gyral frontal lobe	59	21	26	19	5.47
Superior PFC	30	18	65	19	5.10
Superior orbitofrontal cortex	31	15	62	−14	5.49
*YOUNG>OLD*					
Inferior occipital gyrus	29	−42	−82	−23	5.11
Posterior cerebellum	177	42	−79	−23	5.72
Angular gyrus	36	51	−73	31	5.15
Parietal lobe	31	−60	−49	43	5.73
Cingulate gyrus	30	3	−22	43	4.55
Motor cortex	256	48	−4	25	5.65
Caudate	54	−15	11	16	6.21

All coordinates Montreal Neurological Institute. Contrast thresholded with *p*<.0001, and a 25-voxel extent threshold.

Within right hemispheric structures, neural activity was largely equivalent across age groups. The older group showed twice as many significant activations in the left hemisphere as their younger peers. Both groups showed additional motor cortex activation in relation to each other; but the older group exhibited almost twice as many voxel activations in this region as the younger group. The location of activation within brain structures recruited by the two age groups differed. Activation in the cingulate gyrus showed that although both groups recruited this structure when gambling, the younger group showed more *posterior* activation, whereas the older group showed more *anterior* activation.

### Brain region selection: Differences in neuro-vascular function

There were no group differences in beta weight values for precuneus activity (*t*(16) = .225, *p* = .837). This suggests that the group differences in neural activity reported above could reasonably be interpreted as being in relation to the slot machine task and not as a result of age differences in neuro-vasculature.

## Discussion

The key finding in the present study was that older adults recruited a greater number of neural networks from both hemispheres to complete the same gambling task as younger adults. Importantly, the older adults over-recruited several PFC brain structures in relation to younger group, and this included the left superior and orbitofrontal PFC. Although some imaging studies have found reductions in PFC recruitment in older adults when performing tasks [44], [45], this was not the case in the present findings. The data instead more closely fit the compensation model of neuropsychological ageing. In this framework, younger adults recruit the most efficient structures for the task at hand, whereas older adults additionally recruit other regions to maintain performance during the same task, in order to counteract cognitive decline and compensate for neural resources that have become less efficient with age [46].

The finding that older adults over-recruit brain structures during task performance has often also been interpreted as support for the HAROLD model [30], but in the present study only partial support was provided. As noted, one of the central tenets of this model is that while engaged in a cognitive task, older adults demonstrate additional PFC activation in regions *bilateral* to those seen in younger adults. This was not the case in the present study; older adults demonstrated additional activation of prefrontal structures but this was lateralised to the left hemisphere only. It is possible that task-specificity may explain this finding; reduced lateralisation has been found in older adults in the domains of episodic memory, semantic memory, working memory, perception, and inhibitory control among others [30]. The current study suggests while engaged in slot machine play, older adults display additional PFC activity when performing the same task, but that this over-recruitment is limited to the left hemisphere.

These data also highlight an apparent paradox within ageing neuroscience, which is that the brain structures reported to shrink most with ageing (the frontal lobes) are also the regions that often show increased activation during functional tasks. Greenwood [17] frames this phenomenon in terms of the functional plasticity that occurs with cognitive ageing. Functional plasticity refers to the neural progression of altered processing networks in the brain that occurs in parallel with cortical volumetric atrophy in the later stages of life. Greenwood argues that the cognitive deficits experienced as a function of older age generate changes in neural strategy to environmental stimulants, and it is these changes that propel increased activation sites. Certainly, age-related alterations in decision strategies during the Iowa Gambling Task have been identified in the behavioural domain [47]. Wood et al. found that although both young and old adults were successful at solving the task, the younger group relied more on learning and memory mechanisms, whereas the older groups used more emotional-based systems that accurately represented wins and losses.

An alternative explanation is that atrophy in more posterior regions of the brain brought about by age-related amyloid β-protein deposition, triggers additional recruitment of frontal structures. This pathological feature is a major indicator of Alzheimer's Disease and occurs largely in mesial-temporal areas such as the posterior cingulate. There is evidence to suggest that amyloid β-protein deposition can also occur as part of the natural ageing process causing disruption to neural connectivity and in particular the default mode network [48], [49]. It may be the case that in the present study, the occurrence of amyloid β-proteins had functional consequences that redirected activity to more anterior regions such as the PFC. This is in keeping with the earlier preposition that older adults may make more use of emotion-related orbital-frontal networks when gambling compared to their younger peers [47].

Within the behavioural domain executive difficulties among older adults have been shown to predict perseveration in the face of losses – a defining characteristic of pathological gambling [50]. Indeed, older adults report greater gambling problems to the degree that they also exhibit executive difficulties [51]. Thus, slot machines might be a particularly problematic form of gambling for older adults, especially as executive functioning begins to decline. In terms of the current findings, the increased PFC activation evidenced by the older group may be a direct consequence of neural retraining that has shifted emphasis towards new networks near the atrophied areas to facilitate any executive control processes that are required to gamble effectively – a shift that could foreshadow problematic play.

### Limitations and future directions

A caveat was that participants were not permitted to alter the amount wagered per spin. Thus, the degree to which additional activation of neural resources impacts upon wagering decisions could not be assessed. If permitted to do so, older adults with increased PFC activity might have been apt to make less risky responses [32], [52], consistent with the premise that prefrontal structures support executive operations such as inhibitory control processes. Alternatively, greater activation may serve a compensatory function, without which older adults would demonstrate a performance decline [53]. It would thus behoove researchers to assess possible variation in wagering among young and old. Doing so might help explicate how age-related changes in neural activation relate to variations in gambling behaviour. It is also worth noting that although no formal tests of frontal functioning were administered, despite both groups scoring similarly on the test of cognitive functioning, it would be expected that age differences would emerge in tests of executive processes.

In addition, the small sample size meant that while there was sufficient power to detect global over-recruitment on the part of the older adults, more subtle effects such as gender differences may have been present that could not be identified in this study. For example, it has previously been shown in a large sample of elderly adults (*N* = 330) that anatomical age effects may be more apparent for men than women, in the parieto-occipital regions and cerebrospinal fluid volume [54]. Of great interest would be to examine whether functional differences in BOLD response exist between the sexes, and if so, whether this has any bearing on subsequent behavioural measures. Given that men are twice as likely as women to develop problem gambling habits [55], and similar gender effects have been found in both the onset of gambling and the amount spent gambling in older adults [56], larger fMRI studies may be able to shed light on underlying neural activity associated with these behaviours.

### Conclusions

Slot machine gaming is a popular pastime among older adults, and globally has experienced a dramatic growth in recent decades [57]. It is therefore of concern that older adults' gambling habits follow an ascending pattern, and that this age group requires proportionally more referrals to mental health counselling [4]. The present results add to a growing literature identifying differences in the way older and younger adults respond to gambling stimuli, but extend this literature in showing that these differences involve over-recruitment of neural structures, including regions of the PFC.

These data provide preliminary findings that indicate that, in comparison to young adults, older adults over-recruit bilateral and contralateral brain structures and demonstrate increased activation of prefrontal cortices when engaged in slot machine gambling. Future research is now needed to assess whether these age differences in neural activation relate differentially to gambling behaviour and problem gambling in everyday life.
